# Supplementary material for: MetaRibo-Seq measures translation in microbiomes
Source: Nat Commun. 2020 Jun 29;11:3268. doi: 10.1038/s41467-020-17081-z (PMC7324362; doi:10.1038/s41467-020-17081-z)
Supplement: Supplementary file 10 — Supplementary Data 7 [file 41467_2020_17081_MOESM10_ESM.zip › File2/Confidence_High_Taxonomy/83343_out.krona.html]

Javascript must be enabled to view this page.

members
magnitude
magnitudeUnassigned
count
unassigned
taxon
rank

83343\_out

130

2
superkingdom
130

phylum
129
976

129
class
200643

171549
129
order

129
family
171550

239759
genus
129

species
129

SRS011239\_contig\_number\_contig-100\_2315.153919SRS011302\_contig\_number\_23955SRS012969\_contig\_number\_12058SRS013098\_contig\_number\_41171SRS013158\_contig\_number\_1082SRS013476\_contig\_number\_contig-100\_441.104835SRS014412\_contig\_number\_contig-100\_23213.72334SRS014855\_contig\_number\_2120SRS015579\_contig\_number\_contig-100\_769.119776SRS015663\_contig\_number\_19636SRS015694\_contig\_number\_11263SRS015782\_contig\_number\_contig-100\_11672.173215SRS015854\_contig\_number\_18150SRS015960\_contig\_number\_19023SRS016203\_contig\_number\_8628SRS016335\_contig\_number\_contig-100\_7681.42878SRS016954\_contig\_number\_contig-100\_11272.107279SRS017103\_contig\_number\_36477SRS017521\_contig\_number\_48398SRS017701\_contig\_number\_5268SRS017821\_contig\_number\_10558SRS017916\_contig\_number\_9028SRS018351\_contig\_number\_11017SRS018427\_contig\_number\_25592SRS018541\_contig\_number\_7478SRS018817\_contig\_number\_contig-100\_13709.89219SRS018836\_contig\_number\_contig-100\_2362.50908SRS019161\_contig\_number\_contig-100\_8320.52103SRS019445\_contig\_number\_22860SRS019496\_contig\_number\_11805SRS020328\_contig\_number\_13366SRS020394\_contig\_number\_15653SRS021484\_contig\_number\_491SRS022137\_contig\_number\_contig-100\_15920.95182SRS022524\_contig\_number\_10179SRS023971\_contig\_number\_3949SRS024132\_contig\_number\_contig-100\_9482.214058SRS024331\_contig\_number\_23312SRS024388\_contig\_number\_6516SRS024492\_contig\_number\_7603SRS024625\_contig\_number\_contig-100\_14373.72298SRS043701\_contig\_number\_contig-100\_3632.47103SRS043768\_contig\_number\_21321SRS045004\_contig\_number\_22617SRS045645\_contig\_number\_30518SRS045713\_contig\_number\_9506SRS045826\_contig\_number\_136SRS046717\_contig\_number\_9314SRS047014\_contig\_number\_contig-100\_7660.134171SRS049402\_contig\_number\_contig-100\_754.101369SRS049773\_contig\_number\_36387SRS049959\_contig\_number\_29265SRS049995\_contig\_number\_3654SRS050925\_contig\_number\_22622SRS051031\_contig\_number\_16749SRS053398\_contig\_number\_6205SRS053649\_contig\_number\_9944SRS054352\_contig\_number\_3576SRS054905\_contig\_number\_16073SRS055017\_contig\_number\_6862SRS055982\_contig\_number\_6285SRS056259\_contig\_number\_contig-100\_15362.15362SRS056273\_contig\_number\_20070SRS057480\_contig\_number\_contig-100\_5734.51437SRS063190\_contig\_number\_contig-100\_4867.4868SRS065504\_contig\_number\_31161SRS075078\_contig\_number\_14925SRS075773\_contig\_number\_28941SRS075821\_contig\_number\_649SRS075878\_contig\_number\_contig-100\_916.147213SRS075984\_contig\_number\_16780SRS076756\_contig\_number\_contig-100\_1856.107730SRS077024\_contig\_number\_contig-100\_1618.1619SRS077127\_contig\_number\_9220SRS077194\_contig\_number\_23288SRS077231\_contig\_number\_22404SRS077294\_contig\_number\_12319SRS077552\_contig\_number\_contig-100\_3071.149744SRS077641\_contig\_number\_contig-100\_24718.24719SRS077849\_contig\_number\_12290SRS078242\_contig\_number\_21518SRS078419\_contig\_number\_2485SRS097958\_contig\_number\_17915SRS098061\_contig\_number\_8709SRS098571\_contig\_number\_59431SRS098644\_contig\_number\_34066SRS098655\_contig\_number\_10795SRS098717\_contig\_number\_13755SRS098881\_contig\_number\_5457SRS101376\_contig\_number\_5121SRS101433\_contig\_number\_478SRS103987\_contig\_number\_19749SRS1041031\_contig\_number\_contig-100\_17644.97468SRS1041132\_contig\_number\_contig-100\_574.575SRS1041136\_contig\_number\_9461SRS1041139\_contig\_number\_102SRS1041145\_contig\_number\_1410SRS104400\_contig\_number\_507SRS104693\_contig\_number\_9024SRS104975\_contig\_number\_5990SRS105153\_contig\_number\_9098SRS1055022\_contig\_number\_3986SRS140492\_contig\_number\_11188SRS140513\_contig\_number\_4647SRS142542\_contig\_number\_8568SRS142890\_contig\_number\_2366SRS142923\_contig\_number\_contig-100\_3983.53434SRS143372\_contig\_number\_9649SRS143417\_contig\_number\_26909SRS143523\_contig\_number\_17777SRS143895\_contig\_number\_6336SRS143991\_contig\_number\_15107SRS144135\_contig\_number\_29332SRS144297\_contig\_number\_11773SRS144362\_contig\_number\_27642SRS147271\_contig\_number\_contig-100\_10278.156218SRS147346\_contig\_number\_17401SRS147425\_contig\_number\_6644SRS147766\_contig\_number\_contig-100\_9157.32201SRS148196\_contig\_number\_contig-100\_24323.175881SRS148319\_contig\_number\_12056SRS148424\_contig\_number\_27101SRS148721\_contig\_number\_39817SRS148817\_contig\_number\_8476SRS149075\_contig\_number\_11158SRS150029\_contig\_number\_6564SRS892882\_contig\_number\_contig-100\_345.346SRS893369\_contig\_number\_11304SRS893383\_contig\_number\_contig-100\_3706.76688
1118061

1239
phylum
1

1
class
186801

order
1
186802

216572
1
family

459786
genus
1

species
1

SRS144537\_contig\_number\_20172
1897011
